# The relationship between psychological resilience and depression among the diabetes patients under the background of “dynamic zero COVID-19”: the mediating role of stigma and the moderating role of medication burden

**DOI:** 10.3389/fpubh.2023.1124570

**Published:** 2023-08-23

**Authors:** Yujin Mei, Xue Yang, JiaoFeng Gui, YuQing Li, XiaoYun Zhang, Ying Wang, Wenyue Chen, Mingjia Chen, Changjun Liu, Lin Zhang

**Affiliations:** ^1^School of Nursing, Wannan Medical College, Higher Education Park, Wuhu City, Anhui Province, China; ^2^School of Marxism, Jinzhou Medical University, Jinzhou City, Liaoning Province, China; ^3^Department of Internal Medicine Nursing, School of Nursing, Wannan Medical College, Higher Education Park, Wuhu City, Anhui Province, China

**Keywords:** resilience, stigma, medication burden, depression, diabetes patients

## Abstract

**Objective:**

Depression in diabetes patients is caused by their own disease or the surrounding social environment. How to cope with changes in mentality and adjust psychological stress responses, especially under China’s dynamic zero COVID-19 policy, is worth further discussion. The researchers constructed a moderated mediation model to test the effect of psychological resilience during dynamic zero COVID-19 on depression in diabetes patients and the mediating role of stigma and the moderating effect of medication burden.

**Method:**

From June to September, 2022, data were collected in Jinghu District, Wuhu City, Anhui Province, by multi-stage stratified sampling. Firstly, we selected a tertiary hospital randomly in Jinghu District. Secondly, departments are randomly chosen from the hospital. Finally, we set up survey points in each department and randomly select diabetes patients. In addition, we used the Connor-Davidson Elasticity Scale (CD-RISC) to measure psychological resilience of patients, and used the Stigma Scale for Chronic Illness (SSCI) to measure stigma, medication burden was measured by the Diabetes Treatment Burden Scale (DTBQ), and depression was assessed by the Patient Health Questionnaire-9 (PHQ-9). We used SPSS (version 23.0) and PROCESS (version 4.1) for data analysis.

**Results:**

(1) Psychological resilience was negatively correlated with stigma, medication burden, and depression. Stigma was positively associated with medication burden and depression. Medication burden and depression are positively correlated, (2) The mediation analysis showed that psychological resilience had a direct predictive effect on depression, and stigma partially mediated the relationship, and (3) Medication burden moderates the direct pathway by which psychological resilience predicts depression; Medication burden moderates the first half of “psychological resilience → stigma → depression.”

**Conclusion:**

Under the mediating effect of stigma, psychological resilience can improve depression. Medication burden has a moderating effect on the relationship between psychological resilience and depression, and it also has a moderating effect on the relationship between psychological resilience and stigma. These results facilitate the understanding of the relationship mechanisms between psychological resilience and depression.

## 1. Introduction

A health crisis has swept the globe for more than 2 years since the World Health Organization (WHO) officially declared a coronavirus disease (COVID-19) pandemic in 2019 (1). In response to the current situation, the Chinese government has implemented a nationwide “Dynamic Zero COVID-19” policy (2, 3). The policy under the COVID-19 pandemic is also a public health emergency that has had an extremely significant impact on people with diabetes in China (4), as the government has implemented strict control measures requiring people to lock down and stay at home and keep their distance to prevent the spread of the virus (5, 6). Prolonged isolation and uncertainty can easily worsen the psychological condition of people with diabetes (7). Dynamic clearance policies and isolation can easily have a range of consequences for people with diabetes: sudden community closure, chronic and acute stress, fear of infection and concerns about accessing medical care and self-management of blood glucose (8, 9). All these adverse factors may trigger depression (10, 11). People with diabetes are a more susceptible group compared to the normal population (12).

Globally, depression is the second leading cause of disability, characterized by loss of happiness, depressed mood and suicidal behaviors (13). Depression is a common mental illness among people with diabetes and can debilitate and impair the mental health of patients (14). There is a bidirectional association between diabetes and depression, with depression being associated with a 60% increased risk of T2DM as reported in a meta-analysis (15). A prospective study (16) showed a 1.6-fold increase in the prevalence of mild or greater depressive symptoms among older adults with type 2 diabetes and overweight/obesity in the United States from before COVID-19 to the COVID-19 pandemic. Also, in previous studies (17, 18), co-occurrence of psychiatric disorders (e.g., depression) was more prevalent in patients with diabetes than in the general population, with prevalence rates ranging from 15–24% and a prevalence of 12.61 cases of depression per 1,000 patients in the first year after initiation of oral antidiabetic therapy. These results suggest the importance of developing strategies to mitigate the negative impact on the mental health of people with diabetes during COVID-19.

Many factors can affect depression, including psychological resilience. Psychological resilience (PR) plays an important role in adapting to the changes brought about by a pandemic and seeking to restore psychological well-being in people with diabetes (19). PR refers to an individual’s ability or dynamic process to adapt and thrive after a serious threat. Past research (20–22) has reported that PR can buffer t the psychological trauma caused by sudden public health events, depression, negative emotions, and chronic illness. It can also help people with diabetes to increase treatment adherence in the face of challenges and difficulties (23). When people are faced with adversity, traumatic events and post-events, their level of PR plays an important role in maintaining or restoring their psychological well-being. Academic research (24, 25) on the relationship between PR and depression has shown a significant correlation between the two. Martin and Marsh reported (26) that PR was a significant predictor of depression in people with diabetes.

The long-term medication burden of diabetes increases the burden of self-experience for patients, who are left in a state of chronic stress and loss of control, triggering stress reactions including depression, anxiety, stigma and other diabetes-related psychological problems (27). Originally defined as “a characteristic of a person whose reputation has been greatly damaged,” stigma often extends to the psychological stigma that patients experience as a result of certain illnesses (28). As a negative emotion, stigma can have a negative impact on treatment adherence and self-management in people with diabetes (29). Stigma also has a negative impact on person’s mental health, often in the form of depression, anxiety, irritability, etc. (30). A study (31) had shown that PR affected the level of stigma of patients and played an important role in reducing the level of stigma, and the higher level of patient’s PR, the lower level of stigma. Meanwhile, PR can also improve the negative emotional state of patients (32). In the USA (33), resilience is negatively associated with depression and general distress in people with diabetes. For people with diabetes in Germany (34), resilience can be considered a non-specific protective factor against depression, anxiety, and impaired quality of life. The association between resilience and depression has been shown to be strong (35). Lower resilience is also considered a predictor of distress and depression during diabetes (36).

Medication burden (MB) is one of the key components of treatment burden. Xin et al. (37) defined medication burden simply as the burden incurred by patients in obtaining, planning, and organizing medication, taking medication, monitoring treatment, and managing adverse drug reactions. MB can seriously affect patient medication adherence, adverse event rates, readmission rates, prolonged hospital stays and reduced physical function (38, 39), as well as increasing the risk of falls, frailty, and patient mortality (40). At the same time, older patients with diabetes or chronic co-morbidities are more likely to develop various psychological problems during long-term multiple medication use (41), such as negative emotions such as anxiety, distress, worry and depression (42).

**Figure 1:**
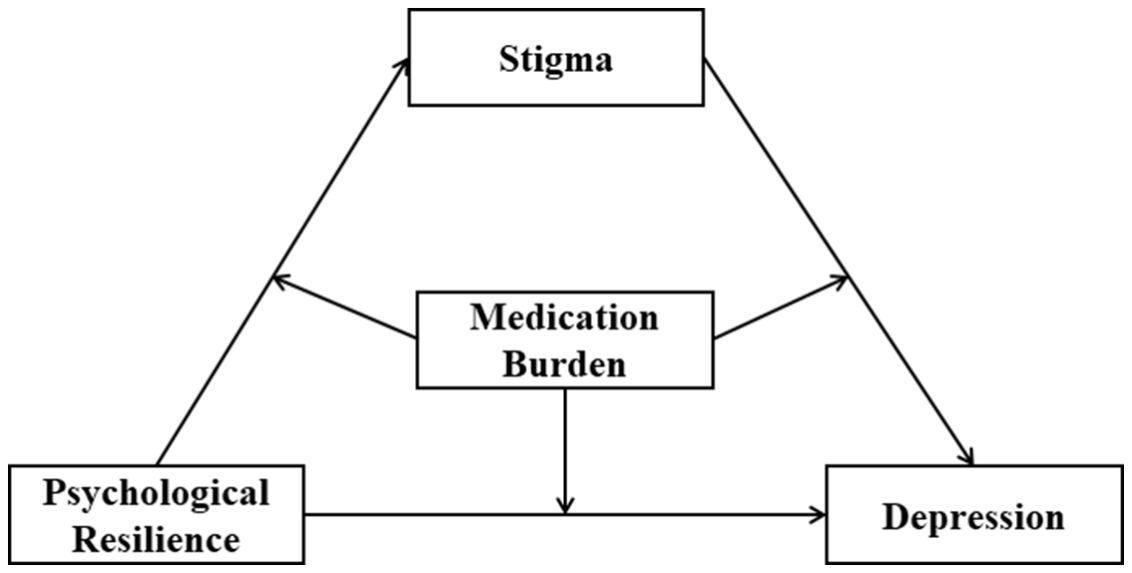
Hypothetical model of the relationships between psychological resilience, stigma, and medication burdenand depression.

Therefore, we hypothesized the following: (1) PR is negatively associated with depression in diabetes patients, (2) Stigma mediates the relationship between PR and depression, and (3) MB plays a moderating role in the relationship between PR, stigma, and depression in diabetes patients (Figure 1).

## 2. Materials and methods

### 2.1. Participants and data collection

Participants were recruited from a tertiary hospital in the Wuhu City (China) by multi-stage sampling. The inclusion criteria were as follows: (i) All patients should meet the diagnostic criteria for DM established by the American Diabetes Association, (ii) Patients are conscious and have full mobility and cognitive ability, and (iii) Patients are willing to cooperate and complete the questionnaire. Exclusion criteria are as follows: (i) presence of severe mental impairment or intellectual problems, (ii) severe diabetic complications or inability to care for themselves, (iii) other serious illnesses such as severe cardiovascular disease, severe infectious diseases, cancer, visual and hearing impairment due to complications of DM, and (iv) pregnancy or other specific diabetes. To reduce errors, relevant personnel was trained before the investigation to clarify communication skills and scoring standards. After obtaining the informed consent of the diabetic, the questionnaire was issued, and the patients answered by themselves. For illiterate patients, the investigators asked face-to-face and then filled out the questionnaire. All methods are implemented following the declaration of Helsinki.

### 2.2. Sampling and sample size

From June to September 2022, this study used a multi-stage sampling method and random number table method to collect data from tertiary hospitals in Jinghu District, Wuhu City, Anhui Province. Firstly, the First Affiliated Hospital of Wannan Medical College was randomly selected from all tertiary hospitals in Wuhu, Anhui Province. Secondly, four departments were strategically selected from this hospital: endocrinology, geriatrics, traditional Chinese medicine, and dermatology. Thirdly, survey points were set up in each department, and diabetes patients were strategically selected for questionnaire surveys. Based on the criteria proposed by Kendall (5 ~ 10 times the number of items and expanded by at least 10% to ensure an adequate sample size), we need at least 228 samples. Eventually, a total of 334 questionnaires were distributed and 329 valid questionnaires were returned, with a valid return rate of 95.85%.

### 2.3. Measurements

#### 2.3.1. Psychological resilience

The Conner-Davidson Resilience Questionnaire (CD-RISC) was developed by psychologists Professors Conner and Davidson in 2003 (43). The CD-RISC contains 25 items on a five-point Likert scale ranging from 0 (“not at all true”) to 4 (“almost always true”). The lower the score, the lower the psychological resilience. The score of the items is added up to the total score of the scale, which is 0 ~ 100 points. The higher the score, the higher the level of psychological resilience. The score ≤ 60 is poor, 61 ~ 69 is average, 70 ~ 79 is good, and ≥ 80 is excellent. The scale consists of 5 dimensions. The first dimension (0 ~ 32 scores) reflects high standards, resilience, and ability. The second dimension (0 ~ 28 scores) reflects dealing with emotions and believing in one’s intuition. The third dimension (0 ~ 20 scores) reflects having a constructive attitude towards change and safe relationships. The fourth dimension (0 ~ 12 scores) is perceived control and the fifth dimension (0 ~ 8 scores) is mental strength. The Cronbach’s alpha value (44) for this study scale was 0.861.

#### 2.3.2. Stigma

The Stigma Scale for Chronic Illness (SSCI) was an instrument developed by Rao to measure stigma in people with chronic illnesses (45). It consists of 24 items and two dimensions: intrinsic stigma (0 ~ 52 scores) and extrinsic stigma (0 ~ 44 scores). The first 13 items refer to internal stigma and ask about the respondent’s own feelings of stigma. The next 11 items ask about the stigma that the respondent feels as a result of external actions. A five-point Likert scale ranging from 0 (never) to 4 (always) was used. The total score is 0 ~ 96, with higher scores indicating greater stigma. The Cronbach’s alpha value (46) for this study scale was 0.829.

#### 2.3.3. Medication burden

The Diabetic Treatment Burden Questionnaire (DTBQ) was compiled by Professors Ishii H and Shin K in 2018 (47), and it is used to assess the burden associated with medication in diabetes patients. The DTBQ includes three factors: administrative burden, (0 ~ 60 score) flexibility burden (0 ~ 18 score), and blood glucose control burden (0 ~ 30 score). A seven-point Likert scale ranging from 0 (strongly disagree) to 6 (strongly agree) was used, the total score is 0 ~ 108. Among them, items of 1 ~ 10 are positive scoring, higher scores indicate a more significant treatment burden; items of 11 ~ 18 are inversely scored, with higher scores indicating a lower treatment burden. The Cronbach’s alpha value (48) for this study scale was 0.780.

#### 2.3.4. Depression

The Patient Health Questionaire-9 (PHQ-9) was derived from the depression part in the Patient Health Questionnaire (PHQ) compiled by Spitzer in 1999 (49). PHQ-9 was recommended by the diagnostic and Statistical Manual of mental disorders, Fifth Edition (DSM-5). The response options for the project range from “not at all” (0 points) to “almost every day” (3 points), the total score is 0 ~ 27. Score 0 ~ 4 with no depression, 5 ~ 9 with mild depression, 10 ~ 14 with moderate depression, 15 ~ 19 with moderate to severe depression, and 20 ~ 27 with severe depression. The scale can not only screen for depression but also show the severity of depression. Because of its convenient use, good reliability, and effectiveness, it has been widely used in medical tumor screening in grass-roots hospitals. The Cronbach’s alpha value (50) for this study scale was 0.842.

### 2.4. Statistical analyses

SPSS23.0 was used to conduct all statistical analyses. Harman single factor test was used for exploratory factor analysis of all the questionnaire items. The results showed that there were 25 factors with eigenvalues greater than 1. The first factor explained only 18.913% of the variance, which was less than 40% critical standard, suggesting that there was no common methodological bias.

We used SPSS 23.0 to accomplish all the statistical analyses. Firstly, we calculated general and controlled variables for descriptive statistics and bivariate correlations. Secondly, we used Hayes’ PROCESS (2013) macro (model 4) to evaluate the mediating effect of stigma. Finally, we analyzed the moderator–mediator model with Hayes’s PROCESS macro (model 8) (2013). All the continuous variables were standardized, and the interaction terms were calculated from these standardized scores. The bootstrap method produces 95% bias-corrected CIs for these effects from 5,000 re-sample of the data. CIs that do not contain zero indicate a significant effect.

### 2.5. Ethical considerations

Approval for this study was given by the medical ethics committee of Wannan Medical College (approval number 2021-3). All the participants were informed that they would bear no penalty for refusal to participate in the study and would be allowed to withdraw at any time without affecting their treatments. Informed consents were obtained before questionnaires were administered to participants.

## 3. Results

### 3.1. Descriptive statistics

Table 1 shows the demographic characteristics of the study objects and a univariate analysis of depression with different features. Of the 329 diabetes patients, 198 (60.2%) were men, and 131 (39.8%) were women. The age range of diabetes patients was 45 ~ 95 years and the mean age being 62.02 ± 11.77 years. There were significant differences in individual monthly income and SMBG. Most patients with diabetes (72.9%) had a middle school education or less. Only 8.5% of diabetes patients were able to SMBG regularly, and more than a third (37.4%) reported a monthly income of less than 1,000 CNY. 7.9% of the older diabetic patients did not have depression. 27.1% of the patients had mild depression; 28.9% of patients were moderately depressed; 27.7% of the patients had moderate to severe depression; 8.4% of older diabetic patients were severely depressed.

**Table 1:** Univariate analysis of depression of diabetic patients with different characteristics (*n* = 329).

Variables	Group	*N* (%)	Mean ± SD	*F/t*	*p*
Age (years)	45 ~ 95	329 (100.0)	62.02 ± 11.77	0.878	0.715
Gender	Male	198 (60.2)	11.81 ± 5.26	0.255	0.614
	Female	131 (39.8)	12.57 ± 5.69		
Education level	Middle school or less	240 (72.9)	12.32 ± 5.34	0.613	0.543
	High or technical secondary school	49 (14.9)	11.63 ± 5.51		
	Junior college or university	40 (12.2)	11.50 ± 5.96		
Monthly income	Less than 1,000 CNY	123 (37.4)	13.55 ± 5.47	7.844	<0.001
	1,000–3,000 CNY	55 (16.7)	11.58 ± 5.61		
	3,000–5,000 CNY	77 (23.4)	12.38 ± 4.62		
	Above 5,000 CNY	74 (22.5)	9.85 ± 5.32		
Course of the disease	< 5 years	101 (30.7)	11.92 ± 5.88	0.345	0.793
	5–10 years	86 (26.1)	12.42 ± 5.04		
	11–20 years	93 (28.3)	11.81 ± 5.04		
	> 20 years	49 (14.9)	12.57 ± 5.97		
Treatment	Take the medicine orally only	150 (45.6)	12.18 ± 5.48	0.700	0.497
	With insulin alone	89 (27.1)	11.58 ± 4.91		
	Medication combined with insulin	90 (27.4)	12.53 ± 5.86		
SMBG	Never monitoring	106 (32.2)	12.88 ± 5.79	4.196	0.016
	No law	195 (59.3)	12.07 ± 5.13		
	Regular monitoring	28 (8.5)	9.57 ± 5.49		
Severe hypoglycemia	Yes	78 (23.7)	12.44 ± 5.04	3.807	0.052
	NO	251 (76.3)	12.02 ± 5.56		

SMBG, self monitor blood glucose.

### 3.2. Bivariate correlation analyses

The mean, standard deviation, and correlation between variables are shown in Table 2. The score for depression was 12.12 ± 5.44. The results indicate that PR has a significant and negatively correlated with stigma (*r* = −0.325, *p* < 0.01) and MB (*r* = −0.243, *p* < 0.01), as well as depression (*r* = −0.391, *p* < 0.01).The stigma was positively correlated with MB (*r* = 0.524, *p* < 0.01) and depression (*r* = 0.590, *p* < 0.01). MB was positively correlated with depression (*r* = 0.267, *p* < 0.01).

**Table 2:** Descriptive statistics and correlations among variables (*n* = 329).

Variables	Mean	SD	1	2	3	4
1 PR	33.73	13.71	1			
2 Stigma	36.22	16.39	−0.325^**^	1		
3 MB	61.64	9.25	−0.243^**^	0.524^**^	1	
4 Depression	12.12	5.44	−0.391^**^	0.590^**^	0.267^**^	1

**p < 0.01; SD, standard deviation; PR, psychological resilience; MB, medication burden.

### 3.3. The mediation analyses

To investigate H1, we examined the mediating effect of stigma on the relationship between PR and depression using the PROCESS 3.3 macro (model 4) proposed by Hayes, after controlling for the variables of monthly income and SMBG demographics (Table 3). The results showed that PR was associated negatively with depression (*β* = −0.069, *p* < 0.001), and PR explained a total of 41.2% of depression (*F* = 56.803, *p* < 0.001, Δ*R*^2^ = 0.412). PR was associated negatively with stigma (*β* = −0.378, *p* < 0.001). Stigma was associated positively with depression (*β* = 0.179, *p* < 0.001). We examined the indirect effect (*β* = −0.068, SE =0.015, 95%CI = [−0.098, −0.039]) and direct effects (*β* = −0.069, SE =0.019, 95%CI = [−0.106, −0.312]) of PR on depression by testing 95% CIs based on 5,000 bootstrapped samples, indicating stigma partially mediated the relationship between PR and depression (Table 4). The indirectly and directly effects accounted for 49.40 and 50.60% of the total effect, respectively.

**Table 3:** Testing the mediation effect of psychological resilience on depression.

Predictors	Stigma				Depression			
	*β*	SE	*t*	95%CI	*β*	SE	*t*	95%CI
Monthly income	1.092	0.748	1.460	−0.380, 2.565	−0.666	0.209	−3.190^**^	−1.078, −0.255
SMBG	−6.421	1.426	−4.503^***^	−9.227, −3.616	0.449	0.409	1.098	−0.356, 1.253
PR	−0.378	0.065	−5.813^***^	−0.507, −0.250	−0.069	0.019	−3.607^***^	−0.106, −0.031
Stigma					0.179	0.015	11.577^***^	0.148, 0.209
*R*^2^	0.161				0.412			
*F*	20.789				56.803			

*p < 0.05; **p < 0.01; ***p < 0.001; SMBG, self monitor blood glucose; PR, psychological resilience.

**Table 4:** Results for effects of psychological resilience on depression with stigma as a mediator.

	Effect	BootSE	BootLLCI	BootULCI	Relative effect size
Indirect effect	−0.068	0.015	−0.098	−0.039	49.4%
Direct effect	−0.069	0.019	−0.106	−0.312	50.6%
Total effect	−0.137	0.022	−0.179	−0.094	100%

### 3.4. The moderation analyses

To examine H2 and H3, we adopted PROCESS macro (Model 8) proposed by Hayes to examine the moderated mediation. Specially, we estimated parameters for two models. In Model 1, we estimated the moderating effect of MB on the relationship between PR and depression. In Model 2, we estimated the moderating effect of MB on the relationship between depression and stigma.

**Figure 2:**
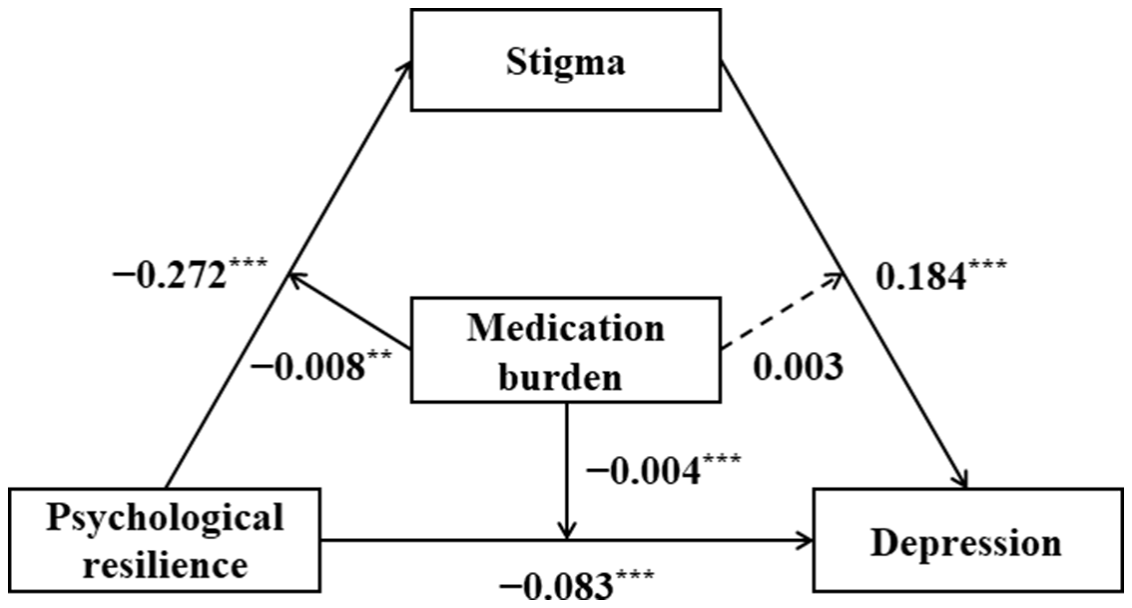
Confirmed model of the relationships between psychological resilience, stigma, and medication burdenand depression.

As shown in Table 5, Model 1 revealed a major impact of PR on stigma (*β* = −0.272, SE = 0.594, 95%CI = [−0.389, −0.156]), which was moderated by MB (*β* = −0.008, SE = 0.003, 95%CI = [−0.148, −0.001]). Model 2 revealed a major impact of PR on depression (*β* = −0.083, SE = 0.019, 95%CI = [−0.121, −0.046]), which was moderated by MB (*β* = −0.004, SE = 0.001, 95%CI = [−0.006, −0.002]). And it revealed a major impact of stigma on depression (*β* = 0.184, SE = 0.017, 95%CI = [−0.149, −0.218]), which was not moderated by MB (*β* = 0.003, SE = 0.001, 95%CI = [−0.006, 0.001]). Hence, hypothesis 2 and 3 were partially supported. The final moderated mediation model was shown in Figure 2.

**Table 5:** Results of the moderated mediation model analysis.

Predictor	Model 1 (Stigma)				Model 2 (Depression)			
	*β*	SE	*t*	95%CI	*β*	SE	*t*	95%CI
Monthly income	0.770	0.658	1.171	−0.524, 2.065	−0.587	0.205	−2.861^**^	−0.990, −0.183
SMBG	−4.641	1.265	−3.669^***^	−7.130, −2.153	0.523	0.398	1.315	−0.259, 1.306
PR	−0.272	0.594	−4.585^***^	−0.389, −0.156	−0.083	0.019	−4.351^***^	−0.121, −0.046
Stigma					0.184	0.017	10.627^***^	0.149, 0.218
MB	0.893	0.092	9.661^***^	0.711, 1.075	0.004	0.033	0.128	−0.060, 0.068
PR × MB	−0.008	0.003	−2.360^**^	−0.148, −0.001	−0.004	0.001	−3.380^***^	−0.006, −0.002
Stigma×MB					0.003	0.001	1.758	−0.001, 0.006
*R*^2^	0.357				0.437			
*F*	35.900				41.605			

*p < 0.05; **p < 0.01; ***p < 0.001; SMBG, self monitor blood glucose; PR, psychological resilience; MB, medication burden.

**Figure 3:**
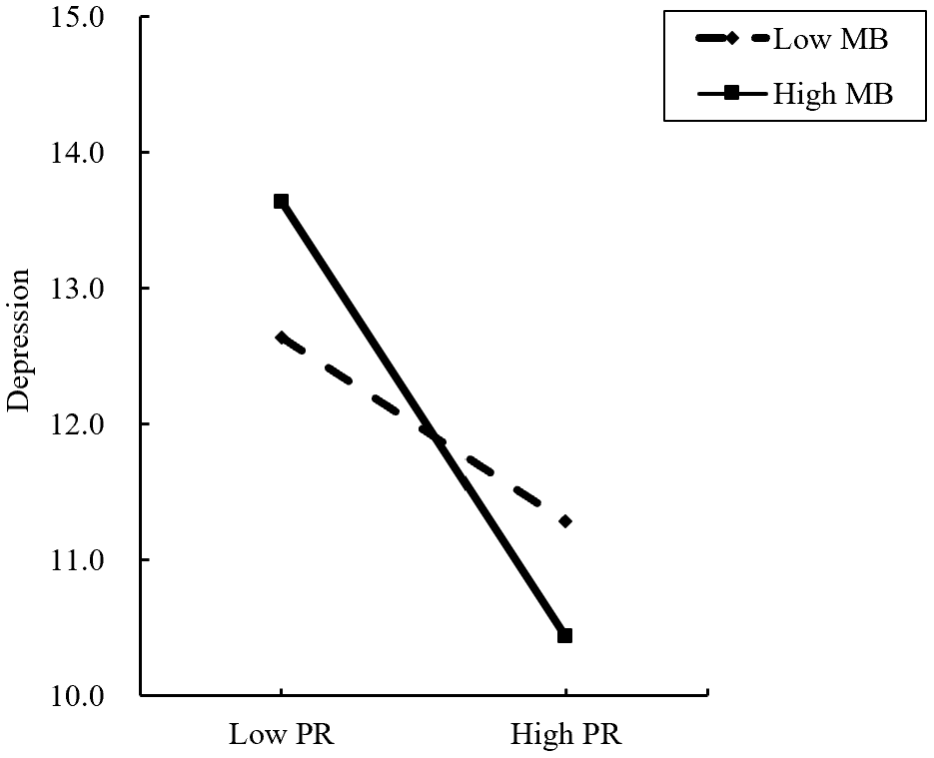
The moderating role of medication burden between psychological resilience and depression.

Figure 3 provides an intuitive view of how the effect of PR on depression is moderated by the MB. A simple slope test shows that for diabetic patients with a low-level MB (*Z* = −1), depression has a significant downward trend with the increase of PR (*β* = −0.049, *p* < 0.001), PR increased by one standard deviation, and the total score of depression decreased by 0.049 standard deviations, that is, the higher the level of PR, the lower the degree of depression; For diabetes patients with high-level MB (*Z* = 1), With the increase of stigma, the degree of depression also showed a significant downward trend (*β* = −0.117, *p* < 0.001), and the downward trend was higher than that of diabetes patients with low MB.

**Figure 4:**
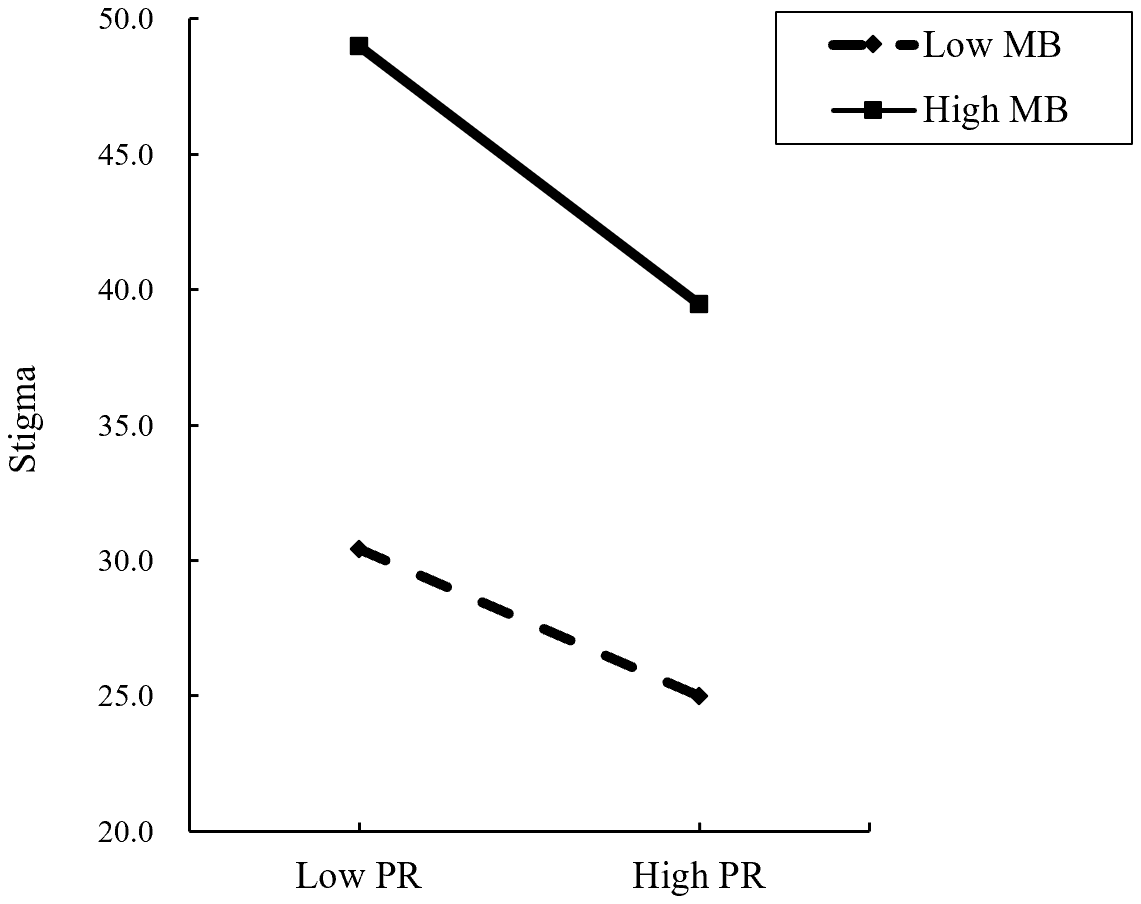
The moderating role of medication burden between psychological resilience and stigma.

Figure 4 shows how the MB moderates the relationship between PR and stigma. A simple slope test showed that for diabetes patients with low-level MB (*Z* = −1), stigma decreased significantly with the increase of PR (*β* = −0.363, *p* < 0.001). One standard deviation increase in psychological resilience was associated with a 0.363 standard deviation decrease in stigma. The higher the PR, the lower the degree of stigma; For diabetes patients with high-level MB (*Z* = 1), with the increase in PR, the decline of stigma was also significant (*β* = −0.639, *p* < 0.001). One standard deviation increase in psychological resilience was associated with a 0.639 standard deviation reduction in stigma, slightly higher than in diabetes patients with a lower MB.

## 4. Discussion

In this study, the results showed that PR was negatively associated with depression in older patients with diabetes, and this relationship was mediated by stigma. Furthermore, the moderating effect suggests that MB moderated these relationships during the COVID-19 policy. And, consistent with our hypothesis about the moderating effect, we found that MB moderated the association between PR and depression, and moderated the effect of PR on stigma. At the same time, the study results showed that only 7.9% of older diabetic patients did not have depression, up to 56% of patients had mild to moderate depression, and 36.1% had moderate to severe depression. This phenomenon indicates that the incidence of depression in older diabetic patients in China is high during COVID-19. Overall, our findings broaden our understanding of PR and depression and contributed to research linking depression to PR.

Older people with diabetes were at high risk for increased depression associated with dangerous public health emergencies. Our results were consistent with previous studies (51, 52), and noted that older adults with diabetes showed a high incidence of depression during the COVID-19 pandemic (53), people going through this period were susceptible to mental health problems (54). Depression could be one of the worst experiences experienced by older people with diabetes, and evidence from different countries suggested that older people with diabetes had the highest increase in rates of psychological distress during the pandemic (55).

Our results showed that PR was negatively correlated with stigma and depression, which was consistent with previous findings (56). Thus, PR and stigma influenced the risk of depression in older people with diabetes due to the COVID-19 pandemic. Patients with higher resilience experience lower levels of stigma and depression because they could cope more successfully with the stressors of the COVID-19 pandemic (57). Research’s (58, 59) had found that PR could help individuals remain optimistic in the face of negative events and improve responses to negative events. Thus, even during the COVID-19 pandemic, compensatory factors such as PR can help offset possible negative effects on mental health (60). Thus, resilience could prevent and improve depression in older people with diabetes during the COVID-19 pandemic.

Modeling predictors of depression had identified PR as a protective factor in young people and stigma as a precipitator of depression. This finding highlighted the importance of monitoring stigma in older diabetics. In our study, stigma was found to mediate between PR and depression, suggesting that older diabetics who experience the shame of their illness and the blame of others reduce perceived PR and thus increase depression in the context of COVID-19. These findings were consistent with previous research on depression (61, 62). Our research suggested that stigma was a harmful negative emotion that reinforces people’s negative feelings about negative events. Therefore, stigma allowed individuals to take a negative rather than a positive view of an event, which could cause them to alter their emotional responses and harm their mental health (63).

Many studies (64, 65) supported MB as a potential regulator. Our findings suggested that MB may play a moderating role in the relationship between loneliness and depression during the COVID-19 pandemic. When people were in a high MB state, people with high PR were less likely to develop depression than people with low PR. MB thus played a significant moderating role by attenuating the perceived relationship between PR and depression. In addition, the impact of MB on PR and stigma varies from individual to individual. When PR level was high, stigma of older diabetic patients with high or low MB showed a decreasing trend. However, the downward trend in older patients with high MB was less than that in patients with low MB. Thus, the results further suggested that elasticity moderated the mediating role of stigma assessment between PR and depression.

In this study, we constructed and tested a hypothetical model based on the results of previous research and theory to determine whether PR might be a protective factor for depression. We also examined the mediating role of stigma and investigated whether MB moderated the mediation model in the context of the COVID-19 pandemic. Clearly, the model shows that older diabetics can maintain their mental health during the COVID-19 pandemic if they have good high levels of mental resilience and pay attention to managing stigma and MB. Resilience is associated with the ability of individuals to manage stress (66). At the same time, psychological problems resulting from continued exposure to traumatic events such as death anxiety and negative emotions remain unavoidable (67). Therefore, PR was one of the most effective strategies that older diabetes could use to solve the negative emotional reactions caused by psychological adversity (68).

The current study suggested that stigma and MB mediate the relationship between PR and depression, a finding that may have useful clinical implications. Our study suggested that the relationship between PR and depression can be partially affected by more frequent stigma in older diabetics. In addition, we found that MB not only moderated the relationship between PR and depression, but also the effect of PR on stigma. Therefore, risk factors such as stigma and MB increased the risk of depression among older Chinese patients with diabetes during the COVID-19 pandemic. These findings suggested that governments and educational institutions should work together to address the deterioration of mental health in older people with diabetes and provide timely and effective services to promote adaptive and positive mental health in this age group. Together, our findings shed light on the relationship between PR and depression and broaden our understanding of how to use protective factors, such as resilience, to formulate public health interventions during the COVID-19 pandemic, especially in the older adult population with diabetes.

### 4.1. Limitations

There are several limitations to this study. Firstly, due to the study’s cross-sectional design, we could not make any causal inferences about the observed associations. Future research should use longitudinal studies to better define the pathways in our theoretical model. Secondly, although self-reporting has been widely used in the literature, this data collection method has inherent disadvantages, such as being highly subjective, inevitably leading to some bias in the data. Future research should include multiple data collection methods to cross-check and obtain more objective and accurate data. Finally, future research should explore the mechanisms of influence of different psychological factors to obtain more accurate findings. Further investigations should not only use a more representative sample and validate the findings, but also focus more on the different mechanisms by which resilience and depression affect others, and clearly measure the impact that the COVID-19 pandemic may have on variables (e.g., depression as a direct result of the pandemic). As a result, future research may involve more psychological structures, such as social anxiety, social support, and self-esteem, to better understand how individuals cope with the adverse consequences of COVID-19 and possibly respond more adaptive in future pandemics.

## 5. Conclusion

This is the first time we have established a moderating mediation model between PR and depression. Our findings showed that stigma played a mediating role in the association between PR and depression. MB plays a role in regulating the relationship between PR and depression. Specifically, with the increase of PR, depression decreased, and the decreasing trend of depression in patients with high MB was greater than that in patients with low MB; In addition, MB also played a moderating role between patient stigma and depression. As patients’ PR increased, stigma decreased, with a slightly greater tendency to decrease in patients with high MB than in those with low MB. Therefore, we should be fully aware of patients’ PR levels and intervene in a timely manner to improve their psychological status. At the same time, MB as an influencing factor needs to be considered with a view to better protecting patients’ psychological well-being. Medical professionals should give regular public lectures on stigma and depression prevention and control measures to achieve early screening, diagnosis, and intervention. Patients should also be regularly questioned about their medication in order to understand the presence of MB and intervene in a timely manner.

## Data availability statement

The raw data supporting the conclusions of this article will be made available by the authors, without undue reservation.

## Ethics statement

The studies involving human participants were reviewed and approved by medical ethics committee of Wannan Medical College. The patients/participants provided their written informed consent to participate in this study.

## Author contributions

LZ conceived and designed the research. YM wrote the paper. YM and LZ analyzed the data. YM, LZ, XY, YL, JG, XZ, YW, and CL revised the paper. YM, XY, JG, YL, XZ, YW, WC, MC, CL, and LZ reviewed the manuscript. All authors contributed to the article and approved the submitted version.

## Funding

The research was supported by the Support Program for Outstanding Young Talents from the Universities and Colleges of Anhui Province for LZ (gxyqZD2021118); The study was supported by the Research on the Creation of International Rules for China’s Participation in Global Health Governance and Path Selection” (21yjcgjw006); The study was supported by the National Innovation and Entrepreneurship Training Program for College Students (202210368016); The study was supported by the Research Practice of Elderly Nursing (2021shsjkc030).

## Conflict of interest

The authors declare that the research was conducted in the absence of any commercial or financial relationships that could be construed as a potential conflict of interest.

## Publisher’s note

All claims expressed in this article are solely those of the authors and do not necessarily represent those of their affiliated organizations, or those of the publisher, the editors and the reviewers. Any product that may be evaluated in this article, or claim that may be made by its manufacturer, is not guaranteed or endorsed by the publisher.

## Abbreviations

CD-RISC: Conner-Davidson Resilience Scale
SSCI: Stigma Scale for Chronic Illness
DTBQ: Diabetes Treatment Burden Scale
COVID-19: Corona Virus Disease 2019
CIs: confidence intervals
DM: Diabetes Mellitus
PHQ-9: Patient Health Questionaire-9
SMBG: Self Monitor Blood Glucose
PR: Psychological Resilience
MB: Medication Burden
ANOVA: One-way Analysis of Variance
